# Structural Insight into the Binding of Cyanovirin-N with the Spike Glycoprotein, M^pro^ and PL^pro^ of SARS-CoV-2: Protein–Protein Interactions, Dynamics Simulations and Free Energy Calculations

**DOI:** 10.3390/molecules26175114

**Published:** 2021-08-24

**Authors:** Devashan Naidoo, Pallab Kar, Ayan Roy, Taurai Mutanda, Joseph Bwapwa, Arnab Sen, Akash Anandraj

**Affiliations:** 1 Centre for Algal Biotechnology, Mangosuthu University of Technology, P.O. Box 12363, Durban 4026, South Africa; mutanda.taurai@mut.ac.za (T.M.); joseph@mut.ac.za (J.B.); Akash@mut.ac.za (A.A.); 2 Bioinformatics Facility, Department of Botany, University of North Bengal, Siliguri 734013, India; pallab_nbu@yahoo.com (P.K.); arnab.sen@nbu.ac.in (A.S.); 3 Department of Biotechnology, Lovely Professional University, Phagwara 144411, India

**Keywords:** SARS-CoV-2, spike protein, M^pro^, PL^pro^, cyanobacteria, cyanovirin-N, scytovirin, phycocyanin, molecular docking, molecular dynamics simulations

## Abstract

The emergence of COVID-19 continues to pose severe threats to global public health. The pandemic has infected over 171 million people and claimed more than 3.5 million lives to date. We investigated the binding potential of antiviral cyanobacterial proteins including cyanovirin-N, scytovirin and phycocyanin with fundamental proteins involved in attachment and replication of SARS-CoV-2. Cyanovirin-N displayed the highest binding energy scores (−16.8 ± 0.02 kcal/mol, −12.3 ± 0.03 kcal/mol and −13.4 ± 0.02 kcal/mol, respectively) with the spike protein, the main protease (M^pro^) and the papainlike protease (PL^pro^) of SARS-CoV-2. Cyanovirin-N was observed to interact with the crucial residues involved in the attachment of the human ACE2 receptor. Analysis of the binding affinities calculated employing the molecular mechanics-Poisson–Boltzmann surface area (MM-PBSA) approach revealed that all forms of energy, except the polar solvation energy, favourably contributed to the interactions of cyanovirin-N with the viral proteins. With particular emphasis on cyanovirin-N, the current work presents evidence for the potential inhibition of SARS-CoV-2 by cyanobacterial proteins, and offers the opportunity for in vitro and in vivo experiments to deploy the cyanobacterial proteins as valuable therapeutics against COVID-19.

## 1. Introduction

**Figure 1:**
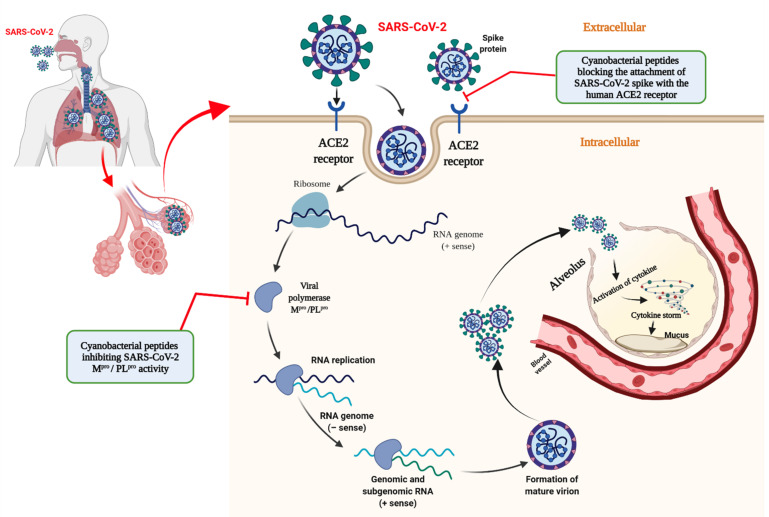
Schematic representation of the rationale for selection of SARS-CoV-2 target proteins, the spike protein, main protease (M^pro^) and papainlike protease (PL^pro^), based on their pivotal roles in cell-entry, infection and replication. The development of the cytokine storm in COVID-19 has also been illustrated; ACE2: Angiotensin-converting enzyme 2. The illustration was created using BioRender.com.

The genome of severe acute respiratory syndrome coronavirus 2 (SARS-CoV-2), associated with the COVID-19 pandemic, encodes several structural and non-structural proteins that play pivotal roles in host attachment, infection and replication [1,2]. SARS-CoV-2 enters and infects the human host through interactions of the viral spike glycoprotein with the host receptor angiotensin-converting enzyme II (ACE2) and the process is mediated by the S1 subunit of the spike protein [3]. Host proteases, including the transmembrane serine protease 2 (TMPRSS2) and cysteine protease cathepsin L (CTSL), play significant roles in activating and priming the spike protein for fusion [4,5,6]. After attachment, the virus enters endosomes and the subsequent cleavage of the spike by TMPRSS2 results in the fusion of viral and lysosomal membranes [7,8]. Respiratory cells, including alveolar and bronchial epithelial cells, expressing ACE2 are the primary targets for infection, although the ACE2 receptor is distributed throughout the human body in tissues including the small intestine, kidneys, heart, liver, colon, etc. [9]. Pulmonary infection with COVID-19 results in mild to moderate or critical illness. In severe cases, SARS-CoV-2 infection causes damage to the alveolar wall leading to increased thrombus burden in pulmonary capillaries leading to acute respiratory distress syndrome [10,11]. Chemokines and cytokines are released in response to an influx of infectious viral particles to establish normal immune responses [12]. Chemokines redirect neutrophils from the bloodstream to alveoli where proinflammatory cytokines including the tumour necrosis factor, interleukin-β, interleukin-6 and interferon gamma (IFN-γ) are produced to neutralize the viral particles [12]. However, excessive levels of proinflammatory cytokines result in the development of an adverse “cytokine storm” that has been correlated with severe illness in COVID-19. Excessive proinflammatory cytokines may result in epithelial and endothelial injury, pneumonia, multiorgan damage, myocarditis, venous thromboembolism and pulmonary embolism [13,14,15,16]. The mechanisms involved in SARS-CoV-2 entry into host cells and replication, and the development of the cytokine storm have been depicted in Figure 1.

The viral replication and transcription complex composed of Nsp2-16 encoded by ORF1a and ORF1b facilitates viral RNA synthesis once SARS-CoV-2 has entered the target cells [17]. Viral replication is achieved through cleavage of polyproteins 1a and 1ab by the papain-like protease (Nsp3; PL^pro^) and the main protease (Nsp5; M^pro^), the process through which Nsp1-16 are released [17]. Structural and non-structural proteins including the spike protein, the M^pro^ and the PL^pro^ represent interesting targets for antiviral drug development. Currently, SARS-CoV-2 infection is treated symptomatically with drugs that have been repurposed. For instance, remdesivir, a broad-spectrum antiviral drug that was developed to treat patients with Ebola and related RNA viruses, has undergone clinical trials after displaying promising results in vitro [18]. A recent clinical trial has reported that the administration of dexamethasone, a potent corticosteroid, to ventilated and non-ventilated patients reduced the number of COVID-19 fatalities by 35% and 20%, respectively [19]. Azithromycin, convalescent plasma therapy and auxiliary blood purification have also been observed to be effective in treating COVID-19 infection [20].

We recently reported on the potential of small molecules from cyanobacteria as promising therapeutic candidates against the M^pro^ and the PL^pro^ of SARS-CoV-2 [21]. In addition to the importance of small molecules in drug development, therapeutic peptide and protein drugs exhibiting molecular weights exceeding 100 kDA have emerged as important medicines against an array of diseases. Therapeutic proteins have been developed to treat various cancers, autoimmune and genetic disorders, inflammation, and infectious diseases [22]. Recently, several peptide inhibitors of SARS-CoV-2 proteins were proposed as therapeutic remedies against COVID-19 and are presently under preclinical evaluation [23,24,25,26]. In the present study, we investigated the potential for the inhibition of the SARS-CoV-2 spike protein, M^pro^ and PL^pro^ [27,28] by antiviral cyanobacterial proteins; cyanovirin-N [29], scytovirin [30] and phycocyanin [31] employing molecular docking approach. The rationale for selecting the inhibitory targets in SARS-CoV-2 has been depicted in Figure 1. Robust molecular dynamics simulations were conducted to validate the results of molecular docking and profile the most promising inhibitory candidates against each of the SARS-CoV-2 proteins, targeted towards the development of effective therapeutics.

## 2. Results and Discussion

### 2.1. Molecular Docking of the Cyanobacterial Proteins with the Receptor-Binding Domain of the SARS-CoV-2 Spike Protein

The spike protein of SARS-CoV-2 attaches to the human receptor angiotensin converting enzyme 2 (ACE2) via the receptor-binding domain (RBD) and paves the foundation for viral entry in host cells [1]. Molecular docking of the cyanobacterial proteins was conducted at the active binding pocket of the receptor binding domain (RBD) of SARS-CoV-2 spike protein (PDB ID: 6LZG), in light of prior knowledge about the active site residues that bind with the human receptor ACE2 [1,32,33]. Detailed binding energy scores have been provided in Table 1. Cyanobacterial proteins cyanovirin-N, scytovirin and phycocyanin exhibited promising binding potential with the RBD of SARS-CoV-2 spike protein and returned binding energy scores of −16.8 ± 0.02 kcal/mol, −12.8 ± 0.01 kcal/mol and −15.1 ± 0.03 kcal/mol (Table 1), respectively.

The spike protein of SARS-CoV-2 forms important protein–protein contacts (PPCs) with residues of the human ACE2 receptor involving large surface areas and deep binding pockets [34,35]. These protein–protein interactions (PPIs) are crucial for viral entry and fusion [36]. Several peptides have now been investigated in silico and, to certain extent, in vitro, for their ability to interact with SARS-CoV-2 proteins. For instance, computational design led to the identification of a 23-mer SBP1 peptide capable of binding the RBD of SARS-CoV-2 with relative conformational stability [25]. A pancoronavirus fusion inhibitor, Ek1 inhibited the SARS-CoV-2 fusion mechanism by binding the HR1 (heptapeptide repeat sequence 1) subunit of the viral spike protein [24]. In the present study, we compared the binding energy scores of the Ek1 peptide and cyanovirin-N protein with the SARS-CoV-2 spike protein. It was interesting to note that the binding energy score of the cyanovirin-N-SARS-CoV-2 spike protein complex (−16.8 ± 0.02 kcal/mol) was significantly higher (*p* < 0.01) than the binding energy value of the Ek1-SARS-CoV-2 spike protein complex (−11.10 ± 0.03 kcal/mol). Furthermore, peptide drugs often exhibit low bioavailability as a consequence of proteolytic degradation and rapid renal elimination [25,37]. These factors have not yet been addressed for the peptides EK1 and SBP1; however, methods to improve the stability and resistance to degradation of cyanovirin-N have already been developed and include the attachment of carbohydrate and polyoxyethylene derivatives [38].

**Figure 2:**
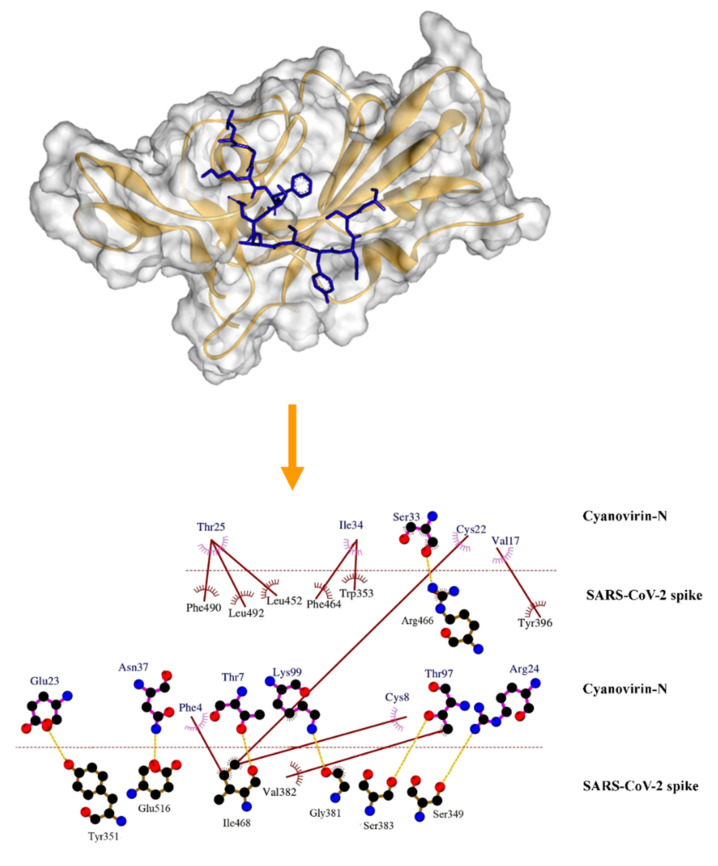
Mode of interaction of the cyanobacterial protein cyanovirin-N with the spike protein of SARS-CoV-2. Yellow ribbon represents the SARS-CoV-2 spike protein. Cyanovirin-N is represented as a blue stick. Hydrophobic interactions have been represented as solid brown lines. Hydrogen bonds have been marked as dashed orange lines.

In the present study, we assessed the binding potential of the cyanovirin-N protein with the SARS-CoV-2 spike protein in comparison with the interaction of human ACE2 receptor-RBD SARS-CoV-2 spike protein complex. It was striking to note that cyanovirin-N displayed significantly higher (*p* < 0.01) binding energy scores than the human ACE2 receptor-RBD-SARS-CoV-2 spike protein complex (−12.5 kcal/mol). Cyanovirin-N was observed to interact with the residues Ser349, Tyr351, Trp353, Gly381, Val382, Ser383, Tyr396, Leu452, Phe464, Arg466, Ile468, Phe490, Leu492 and Glu516 at the active binding pocket of the RBD of SARS-CoV-2 spike protein (Table 1 and Figure 2). The residues Phe490, Leu492 and Glu516 have been reported to be crucial in the attachment of the human ACE2 receptor [34]. Furthermore, the residues Leu455, Phe486, Glu493, Ser494, Asp501 and Tyr505 also play key roles in binding the human ACE2 receptor [32,34].

Cyanovirin-N was first isolated from the cyanobacterium *Nostoc ellipsosporum* and has displayed potent antiviral activity against human immunodeficiency virus (HIV), against which the protein is capable of interrupting viral entry by blocking the envelope glycoprotein-mediated membrane fusion reaction [29]. Affinity chromatography and in vitro analysis revealed that cyanovirin-N interacted with high-mannose oligosaccharide glycans from HIV-gp120 by creating a cross-link between domains A and B of the lectin [39]. In vitro and in vivo experiments later revealed antiviral activity of cyanovirin-N against the Zaire strain of the Ebola virus (ZEBOV) based on its affinity towards envelope glycoproteins on the surface of the Ebola virus [40].

Pathogenic viruses take control of the host’s cellular apparatus to implement their own replication processes, a strategy fortified by glycosylation [41]. The SARS-CoV-2 spike protein undergoes glycosylation to produce glycans that mask polypeptide epitopes and shield it from the innate immune response, and facilitate spike-ACE2 interaction [41,42]. The glycosylation of the SARS-CoV-2 spike protein produces 22 N-linked glycosylation sites that comprise two oligomannose-type, six oligomannose and complex-type and 14 complex-type glycans [43,44]. In SARS-CoV-2, glycans play a significant role at the interface of the spike-ACE2 interaction and are possible drug targets that could reduce binding to ACE2 and subsequent cell–cell transmission [41]. Mazur-Marzec and colleagues comprehensively reviewed the antiviral activities of cyanovirin-N and detailed broad-spectrum activity against viruses including human herpes 6, measles, hepatitis, and the influenza virus, all of which comprise N-linked mannose oligosaccharide glycans [45]. These experimental data, in addition to the computational hypotheses presented herein, generate opportunities for future in vitro and in vivo experiments to assess the inhibitory efficacy of cyanovirin-N against the SARS-CoV-2 spike protein, targeted towards effective drug design.

### 2.2. Molecular Docking of the Cyanobacterial Proteins with the SARS-CoV-2 M^pro^

SARS-CoV-2 replication is arrested by proper inhibition of its vital main protease enzyme M^pro^, which plays a crucial role in the proteolytic cleavage of the viral polyprotein 1ab [35]. Robust molecular docking of the cyanobacterial proteins with the SARS-CoV-2 M^pro^ revealed interesting facts regarding their inhibitory potential. A detailed account of the binding energy scores has been provided in Table 1. The cyanobacterial proteins cyanovirin-N, scytovirin and phycocyanin displayed encouraging binding energy scores of −12.3 ± 0.03 kcal/mol, −9.3 ± 0.02 kcal/mol and −11.0 ± 0.03 kcal/mol, respectively (Table 1).

**Figure 3:**
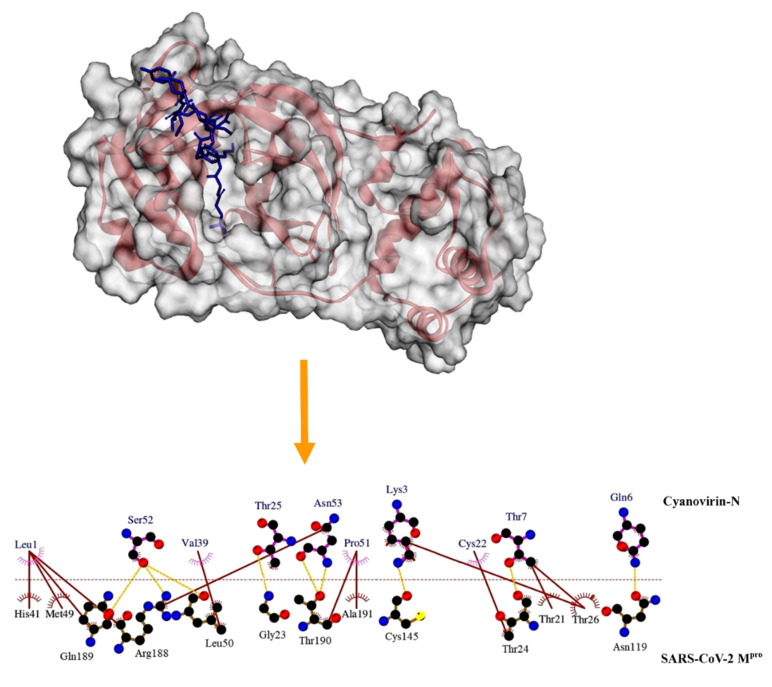
Mode of interaction of the cyanobacterial protein cyanovirin-N with the SARS-CoV-2 M^pro^. Red ribbon represents the SARS-CoV-2 M^pro^. Cyanovirin-N has been represented as a blue stick. Hydrophobic interactions have been represented as solid brown lines. Hydrogen bonds have been marked as dashed orange lines.

The binding efficacies of the cyanobacterial proteins with SARS-CoV-2 M^pro^ were further compared with the interaction profile of the Michael acceptor (peptidyl) inhibitor that has been recently reported to exhibit significant inhibitory efficacy against the SARS-CoV-2 M^pro^ [37]. Quite interestingly, the cyanobacterial proteins displayed significantly higher (*p* < 0.01) binding energy scores than the Michael acceptor (peptidyl) inhibitor-SARS-CoV-2 M^pro^ complex (−7.1 kcal/mol) (Table 1) [27]. Furthermore, the electrophilicity of α and β unsaturated systems suggests that the Michael acceptor may interact with off-target proteins [46]. The Michael acceptor was deployed based on its specificity towards cysteine proteases since covalent bonding with Cys145 ensured the inhibition of catalytic activity. Cyanovirin-N was noted to show the highest binding energy score among the cyanobacterial proteins and interacted with the crucial residues of the catalytic dyad including Cys145 and His41. The interaction of cyanovirin-N with Cys145 involved a hydrogen bond with Lys3 while a hydrophobic interaction between His41 of the M^pro^ and Leu1 of the inhibitor aided in binding the protein (Figure 3). Active site residues including Thr21, Gly23, Thr24, Thr26, Met49, Leu50, Asn119, Arg188, Gln189, Thr190 and Ala191 were also points of contact between cyanovirin-N and the SARS-CoV-2 M^pro^ protein (Table 1 and Figure 3). In addition to the importance of Cys145 and His41, residues Gln189, Thr190 and Ala191 also play key roles in enzymatic activity by linking domains II and III of the main protease [35]. The interactions of cyanovirin-N with integral residues including Met49 located within the hydrophobic subsite (S2) and Thr 24 of promoter A, provide structure to the complex. Thus, the cyanobacterial proteins showed promising binding potential (in terms of binding energy) with the SARS-CoV-2 M^pro^ and offer scope to be further explored in vitro and in vivo to articulately infer their inhibitory potential.

### 2.3. Molecular Docking of the Cyanobacterial Proteins with the SARS-CoV-2 PL^pro^

**Figure 4:**
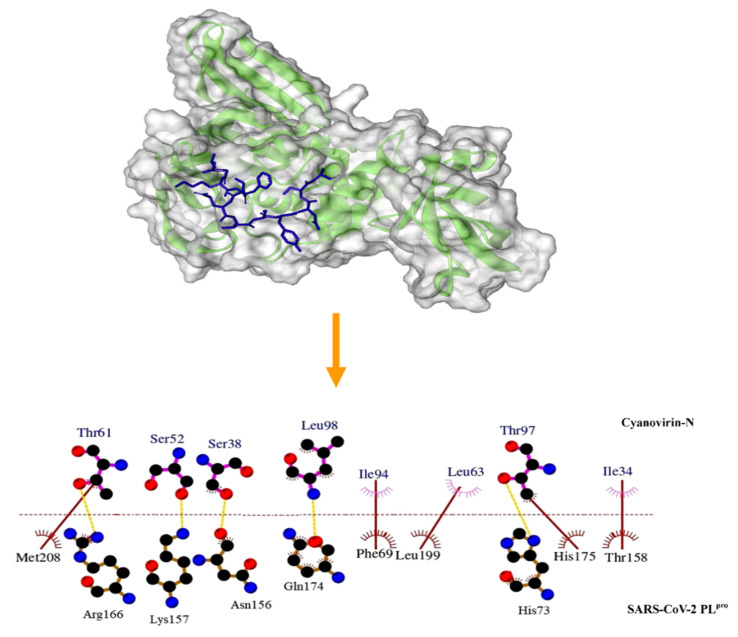
Mode of interaction of the cyanobacterial protein cyanovirin-N with the SARS-CoV-2 PL^pro^. Green ribbon represents the SARS-CoV-2 PL^pro^. Cyanovirin-N has been represented as a blue stick. Hydrophobic interactions have been represented as solid brown lines. Hydrogen bonds have been marked as dashed orange lines.

The PL^pro^ has been reported to be associated with essential mechanisms involved in processing and cleavage of viral polyproteins that facilitate the spread of the virus [47]. In addition, in consequence of the alterations present in the genome of SARS-CoV-2, the PL^pro^ is now well-equipped to cleave the ubiquitinlike interferon-stimulated gene 15 protein [47], thereby evading the host’s innate immune responses. In the present study, we performed an extensive molecular docking of the cyanobacterial proteins at the active binding pocket of the SARS-CoV-2 PL^pro^. The binding energy scores have been depicted in Table 1. The cyanobacterial proteins cyanovirin-N, scytovirin and phycocyanin returned strong binding energy scores of −13.4 ± 0.02 kcal/mol, −10.9 ± 0.01 kcal/mol and −12.6 ± 0.02 kcal/mol, respectively, with the SARS-CoV-2 PL^pro^. Cyanovirin-N displayed the highest binding energy score and was noted to interact with the active site residues Phe69, His73, Thr74, His89, Lys92, Asn156, Lys157, Thr158, Glu161, Asp164, Arg166, Gln174, His175, Leu199, Gln203, Met208 and Gln269 of the SARS-CoV-2 PL^pro^ (Table 1 and Figure 4).

We further explored the binding potential of the cyanobacterial proteins with SARS-CoV-2 PL^pro^ in light of the interaction profile of the recently proposed peptide inhibitors VIR250 and VIR251 [48]. It was interesting to note that the cyanobacterial proteins displayed significantly higher (*p* < 0.01) binding energy scores than the peptide inhibitors VIR250 (−7.0 kcal/mol) and VIR251 (−6.9 kcal/mol) (Table 1). The present findings offer opportunities to exploit the inhibitory potential of the cyanobacterial proteins against SARS-CoV-2 PL^pro^ in vitro and in vivo.

### 2.4. Analysis of Molecular Dynamics Simulations of the Protein–Protein Complexes

**Figure 5:**
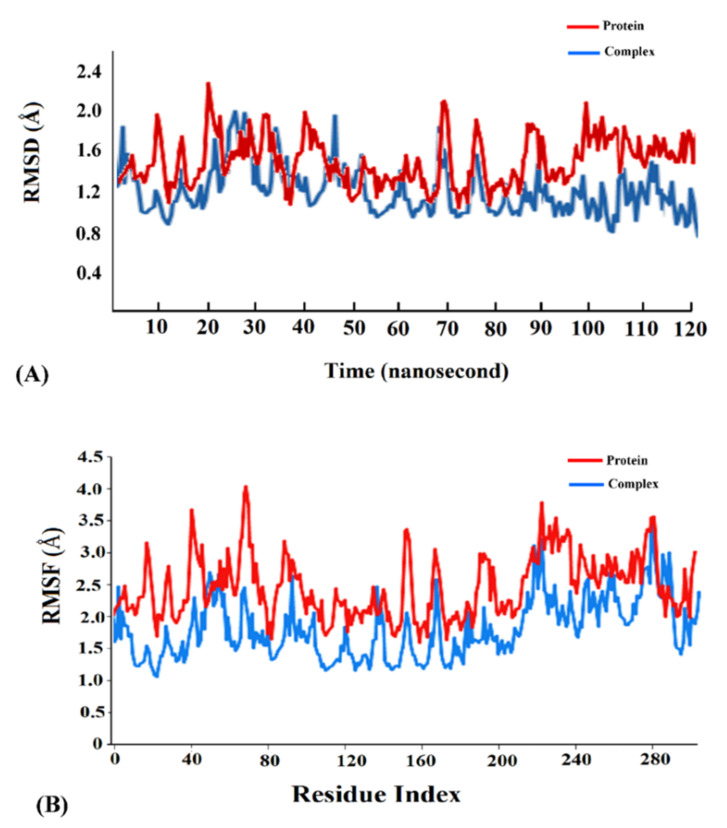
(**A**) Root mean square deviation (RMSD) trajectories of the free receptor SARS-CoV-2 spike protein (red) and the cyanovirin-N-SARS-CoV-2 spike protein complex (blue). (**B**) Root mean square fluctuation (RMSF) trajectories of the free receptor SARS-CoV-2 spike protein (red) and the cyanovirin-N-SARS-CoV-2 spike protein complex (blue).

**Figure 6:**
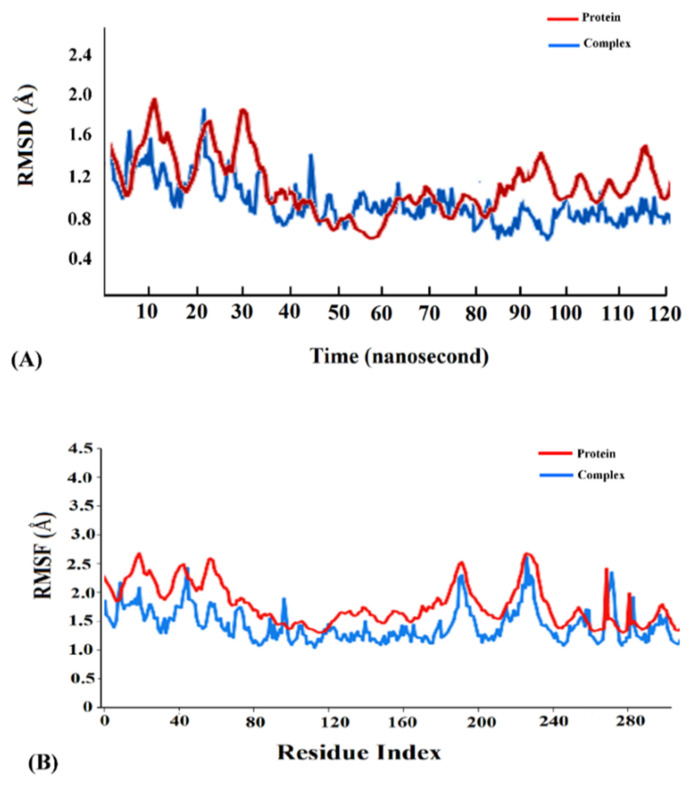
(**A**) Root mean square deviation (RMSD) trajectories of the free receptor SARS-CoV-2 M^pro^ (red) and the cyanovirin-N-SARS-CoV-2 M^pro^ complex (blue). (**B**) Root mean square fluctuation (RMSF) trajectories of the free receptor SARS-CoV-2 M^pro^ (red) and the cyanovirin-N-SARS-CoV-2 M^pro^ complex (blue).

**Figure 7:**
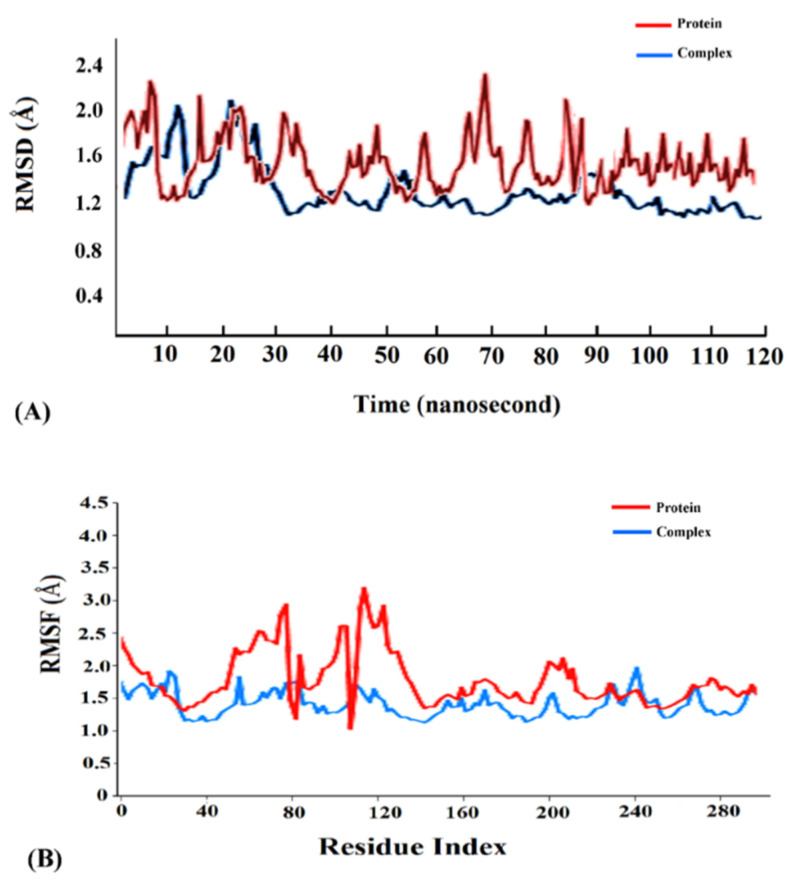
(**A**) Root mean square deviation (RMSD) trajectories of the free receptor SARS-CoV-2 PL^pro^ (red) and the cyanovirin-N-SARS-CoV-2 PL^pro^ complex (blue). (**B**) Root mean square fluctuation (RMSF) trajectories of the free receptor SARS-CoV-2 PL^pro^ (red) and the cyanovirin-N-SARS-CoV-2 PL^pro^ complex (blue).

Cyanovirin-N was found to display the highest binding energy scores with the SARS-CoV-2 spike, M^pro^ and PL^pro^ proteins among the cyanobacterial proteins (Table 1). Accordingly, the cyanovirin-N-SARS-CoV-2 spike, cyanovirin-N-SARS-CoV-2 M^pro^ and cyanovirin-N-SARS-CoV-2 PL^pro^ complexes were chosen for molecular dynamics (MD) simulations for a timescale of 120 ns to validate the results of molecular docking and assess the conformational stability of the complexes. A thorough RMSD analysis along the timescale of 120 ns revealed that the complexes were conformationally stable. A detailed RMSD analysis of the cyanovirin-N-SARS-CoV-2 spike complex revealed fluctuations in RMSD values of Cα atoms until 108 ns and attained stability thereafter (Figure 5A). The cyanovirin-N-SARS-CoV-2 M^pro^ and cyanovirin-N-SARS-CoV-2PL^pro^ complexes also showed fluctuations in the RMSD values of Cα atoms during the initial phases of MD simulations and attained stability after 110 ns and 103 ns, respectively (Figure 6A and Figure 7A). The average RMSD values of the cyanovirin-N-SARS-CoV-2 spike, cyanovirin-N-SARS-CoV-2 M^pro^ and cyanovirin-N-SARS-CoV-2 PL^pro^ complexes were observed to be 1.5 Å, 0.9 Å and 1.3 Å, respectively (Figure 5A, Figure 6A and Figure 7A). On the contrary, the free spike, M^pro^ and PL^pro^ proteins were noted to display considerable fluctuations throughout the timescale of the MD simulations and returned average RMSD values of 1.9 Å, 1.5 Å and 1.8 Å, respectively (Figure 5A, Figure 6A and Figure 7A). It has been suggested that lower RMSD values reflect higher conformational stability of protein–protein complexes [22,27]. The present findings reflected the decent binding potential of cyanovirin-N with the SARS-CoV-2 proteins and emphasized the fact that the binding of cyanovirin-N to the viral proteins imparted considerable stability to the complexes [21].

Analysis of root mean square fluctuation (RMSF) of the residues of a protein–protein complex is instrumental in exploring the local changes in a protein chain and assessing its stability on binding another protein [21,49]. A detailed RMSF analysis of the cyanovirin-N-SARS-CoV-2 spike, cyanovirin-N-SARS-CoV-2 M^pro^ and cyanovirin-N-SARS-CoV-2 PL^pro^ complexes with respect to the free SARS-CoV-2 spike, M^pro^ and PL^pro^ proteins revealed comparatively lower fluctuations of the complexes compared to the free proteins (Figure 5B, Figure 6B and Figure 7B), thus signifying higher stability of the complexes. The average RMSF values of the cyanovirin-N-SARS-CoV-2 spike, cyanovirin-N-SARS-CoV-2 M^pro^ and cyanovirin-N-SARS-CoV-2 PL^pro^ complexes were found to be 2.1 Å, 1.4 Å and 1.1 Å, respectively, whereas the average RMSF values of the free SARS-CoV-2 spike, M^pro^ and PL^pro^ proteins were observed to be 3.4 Å, 2.2 Å and 1.9 Å, respectively (Figure 5B, Figure 6B and Figure 7B). The peaks in the RMSF plot refer to the residues that experience major fluctuations during the MD simulations [49]. It was evident from the RMSF analysis of the concerned protein–protein complexes that the residues in the active binding pockets of the viral proteins that interacted with cyanovirin-N (Table 1) were relatively stable (Figure 5B, Figure 6B and Figure 7B) throughout the course of the MD simulations of 120 ns and thus, pointed towards the conformational stability of the complexes.

### 2.5. Intermolecular Hydrogen Bonds in the Protein–Protein Complexes

**Figure 8:**
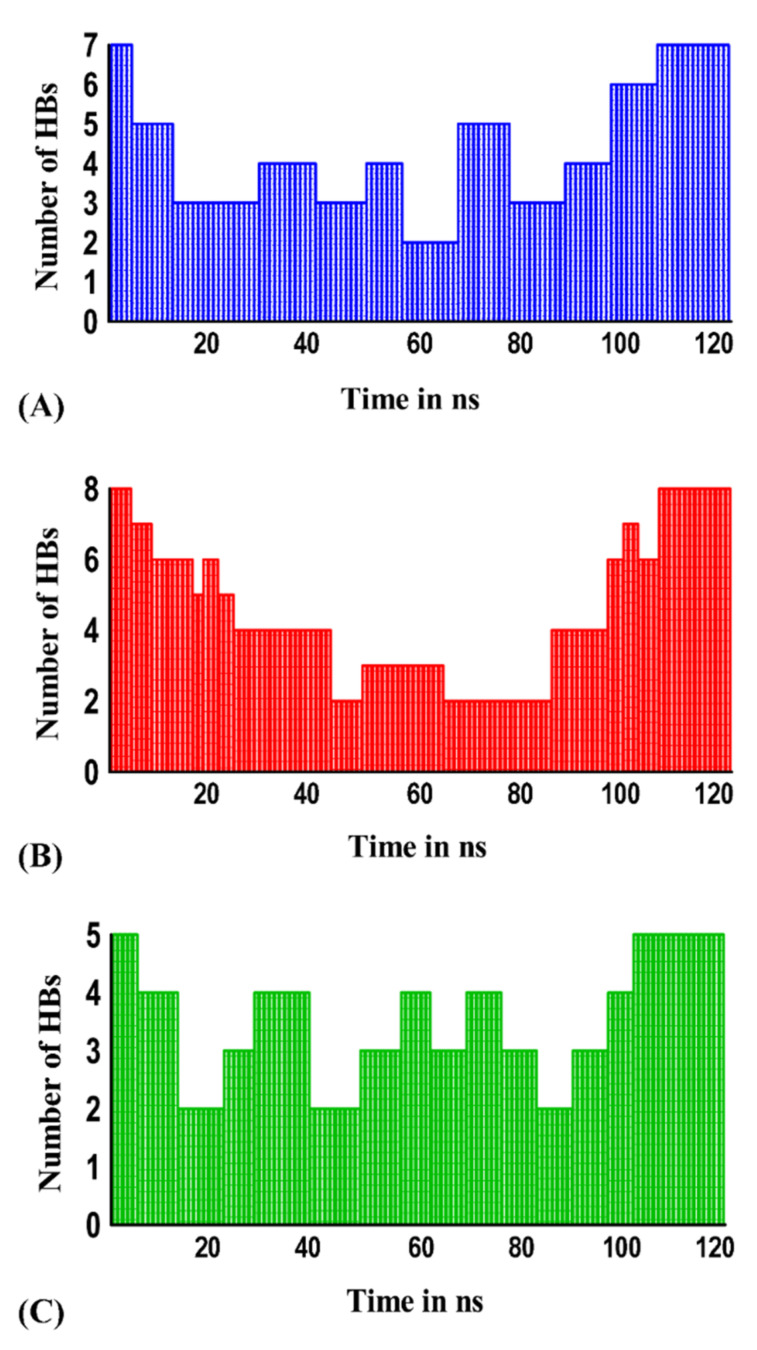
Number of hydrogen bonds in (**A**) cyanovirin-N-SARS-CoV-2 spike protein complex (blue) as a function of time during MD simulations of 120 ns (**B**) cyanovirin-N-SARS-CoV-2 M^pro^ complex (red) as a function of time during MD simulations of 120 ns (**C**) cyanovirin-N-SARS-CoV-2 PL^pro^ complex (green) as a function of time during MD simulations of 120 ns.

Intermolecular hydrogen bonds (H-bonds) impart stability to a protein–protein complex [21,50]. The assessment of H-bonds in a molecular complex during MD simulations aids in to understanding the stability of the complex [50]. A thorough analysis of the hydrogen bonding trajectories associated with the MD simulations of the cyanovirin-N-SARS-CoV-2 spike, cyanovirin-N-SARS-CoV-2 M^pro^ and cyanovirin-N-SARS-CoV-2 PL^pro^ complexes revealed that they did not experience any change in the number of H-bonds prior to and after the MD simulations of 120 ns. Though the complexes experienced fluctuations during the initial phases of MD simulations and displayed a minimum of 2 H-bonds each, there was a gradual increase in the number of H-bonds (Figure 8A–C). The cyanovirin-N-SARS-CoV-2 spike, cyanovirin-N-SARS-CoV-2 M^pro^ and cyanovirin-N-SARS-CoV-2 PL^pro^ complexes displayed a maximum number of 7, 8 and 5 H-bonds, respectively (Figure 8A–C) at the end of 120 ns MD simulation which was in accordance with the number of H-bonds prior to MD simulations. Thus, it was evident from the H-bond analysis that the respective protein–protein complexes were stable.

### 2.6. Analysis of MM-PBSA Free Energies of Binding of the Protein–Protein Complexes

The cyanovirin-N-SARS-CoV-2 spike, cyanovirin-N-SARS-CoV-2 M^pro^ and cyanovirin-N-SARS-CoV-2 PL^pro^ complexes were selected for further estimation of the free energies of binding using the molecular mechanics-Poisson–Boltzmann surface area (MM-PBSA) method. It has been suggested that MM-PBSA provides accurate estimates of free energies of binding of protein–protein complexes and a more negative value indicates stronger binding [51]. The detailed information regarding the electrostatic energy, SASA (Solvent Accessible Surface Areas) energy, van der Waals energy, polar solvation energy and final binding energy from 20 ns to 120 ns with 20 ns interval has been detailed in Table 2. It was evident that all forms of energy, except the polar solvation energy, favourably contributed to the interactions of the cyanovirin-N with the viral proteins (Table 2). The cyanovirin-N-SARS-CoV-2 spike, cyanovirin-N-SARS-CoV-2 M^pro^ and cyanovirin-N-SARS-CoV-2 PL^pro^ complexes displayed final binding energy scores of −84.86 ± 1.08 kcal/mol, −62.13 ± 1.14 kcal/mol and −67.69 ± 1.10 kcal/mol, respectively (Table 2) which signified considerable conformational stability of the complexes after the MD simulations for 120 ns.

## 3. Materials and Methods

### 3.1. Molecular Docking of the Cyanobacterial Proteins with the SARS-CoV-2 Proteins

The high-resolution X-ray diffraction crystal structures of the spike protein (PDB ID: 6LZG Chain B, 2.50 Å resolution), M^pro^ (PDB ID: 6LU7 Chain A; 2.16 Å resolution) and PL^pro^ (PDB ID: 6W9C Chain A; 2.70 Å resolution) proteins of SARS-CoV-2 were downloaded from the PDB. The cyanobacterial proteins of interest were also retrieved from the PDB and these included cyanovirin-N (PDB ID: 3EZM Chain A; 1.50 Å resolution), scytovirin (PDB ID: 2JMV Chain A; 2.0 Å resolution) and phycocyanin (PDB ID: 1I7YChain A; 2.50 Å resolution). The respective viral and cyanobacterial protein structures were prepared for docking analysis by removing the associated water and ligand molecules. Polar hydrogen atoms and Kollman charges were added to the structures using AutoDock tools. The process of energy minimization was achieved using Gromos 96 force field. The refined protein structures were subjected to molecular docking using the GRAMMX server [52] in light of the existing information regarding the active interaction/inhibition binding pockets of the respective viral proteins [18,32,53,54]. The binding energy scores of the respective protein–protein complexes were estimated using the PRODIGY HADDOCK server [55]. The complexes with the lowest binding energy scores and RMSD < 2.0 Å [21] were used for further interaction analysis using the Dimplot software [56].

### 3.2. Molecular Dynamics Simulations

Molecular dynamics (MD) simulation is an essential step in validating the results of molecular docking and helps to accurately assess the potential stability of protein–ligand/protein–protein complexes [27]. Antiviral cyanobacterial proteins that displayed the most encouraging interaction scores (in terms of binding energy values) with each SARS-CoV-2 protein were subjected to MD simulation for a timescale of 120 nanoseconds (ns) using GROMACS software (version 2019) [57]. The MD simulations were executed as per the protocol opted by Khan and colleagues [58]. The equilibration steps were set with constant pressure and temperature (NPT) ensemble [27]. A standard temperature of 300 K and pressure level of 1.013 bar was employed for the simulation process [27]. The MD simulation parameters including the root mean square deviation (RMSD), the root mean square fluctuation (RMSF) and the hydrogen bonds of the respective protein–protein complexes were estimated as a function of time (120 ns) to assess their conformational and structural stability [58].

### 3.3. Estimation of Free Energies of Binding Employing Molecular Mechanics-Poisson–Boltzmann Surface Area (MM-PBSA) Method

Assessment of molecular mechanics-Poisson–Boltzmann surface area (MM-PBSA)-based free energies of binding of protein–ligand/protein–protein complexes in combination with MD simulations provides an apt estimate of the conformational stabilities of protein–ligand/protein–protein complexes [59]. MM-PBSA-based free energies of binding of the selected protein–protein complexes were estimated employing the ‘g_mmpbsa’ script of GROMACS [60].

## 4. Conclusions

The COVID-19 pandemic, with its rapid transmission and infectivity, has been a global calamity. The current work investigated the binding prospects of the known antiviral proteins from cyanobacteria with the crucial proteins of SARS-CoV-2. Interestingly, all the cyanobacterial proteins, namely, cyanovirin-N, scytovirin and phycocyanin, employed in the present study, displayed high binding energy scores with the SARS-CoV-2 proteins. Robust molecular dynamics simulations and a thorough analysis of the binding free energies based on MM-PBSA method established cyanovirin-N as the most promising inhibitor against the imperative SARS-CoV-2 spike, M^pro^ and PL^pro^ proteins. Present research findings offer ample scopes to further exploit the potential of these antiviral cyanobacterial proteins as successful inhibitors of SARS-CoV-2 and bolster the global efforts towards the development of novel effective therapeutics against COVID-19.

## Figures and Tables

**Table 1:** Interaction profile of antiviral cyanobacterial proteins against the SARS-CoV-2 proteins.

SARS-CoV-2 Target Protein	Cyanobacterial Protein	Binding Energy Score (kcal/mol)	Interacting Residues of SARS-CoV-2 Protein
Spike	Cyanovirin-N	−16.8 ± 0.02	*Trp353*, *Val382*, *Tyr396*, *Leu452*, *Phe464*, *Phe490*, *Leu492*, **Ser349**, **Tyr351**, **Gly381**, **Ser383**, **Arg466**, **Glu516**, ***Ile468***
	Scytovirin	−12.8 ± 0.01	*Ala348*, *Ala352*, *Ile468*, **Thr345**, **Arg346**, **Arg357**, **Thr470**
	Phycocyanin	−15.1 ± 0.03	*Tyr380*, *Phe429*, **Gly381**, **Arg408**, **Asp428**, **Leu517**
M^pro^	Cyanovirin-N	−12.3 ± 0.03	*Thr21*, **Gly23**, ***Thr24***, *Thr26*, *His41*, *Met49*, **Asn119**, **Cys145**, ***Leu50***, ***Arg188***, ***Gln189***, ***Thr190***, *Ala191*,
	Scytovirin	−9.3 ± 0.02	*His41*, *Cys44*, *Thr45*, *Met49*, *Leu50*, *Cys145*, *Pro168*, **Thr25**, **Ser40**, **Asn142**, **Glu166**
	Phycocyanin	−11.0 ± 0.03	*Thr45*, *Met49*, *His164*, *Met165*, *Pro168*, *Thr190*, *Ala191*, **Thr24**, **His41**, **Asn142**, **Cys145**
PL^pro^	Cyanovirin-N	−13.4 ± 0.02	*Phe69*, **His73**, *Thr158*, *His175*, *Leu199*, **Asn156**, **Lys157**, **Arg166**, **Gln174**, *Met208*
	Scytovirin	−10.9 ± 0.01	*His255*, *Thr257*, *Thr259*, *Tyr305*, *Tyr310*, *Thr313*, **Gln121**, **Lys279**, **Glu307**, **Ser309**
	Phycocyanin	−12.6 ± 0.02	*Leu190*, *Thr197*, *Val202*, *Met208*, *Thr210*, *Val220*, *Ile222*, *Thr225*, *Ala246*, **Ser170**, **Lys232**

Hydrophobic interactions are represented by italicized text while hydrogen bonding is represented in bold text.

**Table 2:** Details of the binding free energy (±standard deviation) for cyanovirin-N-SARS-CoV-2 spike, cyanovirin-N-SARS-CoV-2 M^pro^ andcyanovirin-N-SARS-CoV-2 PL^pro^ complexes calculated using the MM-PBSA method from 20 to 120 ns with 20 ns interval during the MD simulations.

Complexes	Time (ns)	Van der Waals Energy (kcal/mol)	SASA Energy (kcal/mol)	Electrostatic Energy (kcal/mol)	Polar Solvation Energy (kcal/mol)	Binding Energy (kcal/mol)
Cyanovirin-N-SARS-CoV-2 spike complex	20	−81.42 ± 2.56	−7.35 ± 0.53	−17.43 ± 0.57	33.14 ± 1.47	−73.06 ± 2.19
	40	−87.73 ± 2.33	−7.73 ± 0.42	−18.04 ± 0.41	34.60 ± 1.32	−78.90 ± 1.84
	60	−90.52 ± 2.21	−7.97 ± 0.33	−18.61 ± 0.32	35.56 ± 1.21	−81.54 ± 1.65
	80	−91.90 ± 2.11	−8.10 ± 0.27	−19.02 ± 0.24	36.25 ± 1.12	−82.77 ± 1.50
	100	−93.44 ± 1.79	−8.22 ± 0.19	−19.23 ± 0.19	36.94 ± 1.01	−83.95 ± 1.16
	120	−94.52 ± 1.67	−8.30 ± 0.12	−19.49 ± 0.08	37.45 ± 0.79	−84.86 ± 1.08
Cyanovirin-N-SARS-CoV-2 protease M^pro^ complex	20	−59.61 ± 2.55	−5.38 ± 0.51	−12.76 ± 0.62	24.26 ± 1.39	−53.49 ± 2.29
	40	−64.23 ± 2.31	−5.66 ± 0.41	−13.21 ± 0.55	25.33 ± 1.22	−57.77 ± 2.05
	60	−66.27 ± 2.27	−5.83 ± 0.35	−13.62 ± 0.42	26.04 ± 1.19	−59.68 ± 1.85
	80	−67.29 ± 2.19	−5.93 ± 0.29	−13.93 ± 0.31	26.54 ± 1.02	−60.61 ± 1.77
	100	−68.41 ± 1.92	−6.02 ± 0.22	−14.08 ± 0.27	27.04 ± 0.95	−61.47 ± 1.46
	120	−69.20 ± 1.73	−6.08 ± 0.12	−14.27 ± 0.13	27.42 ± 0.84	−62.13 ± 1.14
Cyanovirin-N-SARS-CoV-2 PL^pro^ complex	20	−64.95 ± 2.59	−5.87 ± 0.69	−13.90 ± 0.61	26.43 ± 1.39	−58.29 ± 2.50
	40	−69.97 ± 2.42	−6.17 ± 0.57	−14.39 ± 0.52	27.60 ± 1.31	−62.93 ± 2.20
	60	−72.20 ± 2.31	−6.36 ± 0.42	−14.84 ± 0.43	28.37 ± 1.23	−65.03 ± 1.93
	80	−73.30 ± 2.18	−6.46 ± 0.33	−15.17 ± 0.32	28.92 ± 1.16	−66.01 ± 1.67
	100	−74.53 ± 1.74	−6.55 ± 0.21	−15.34 ± 0.23	29.46 ± 1.04	−66.96 ± 1.14
	120	−75.39 ± 1.59	−6.62 ± 0.15	−15.55 ± 0.18	29.87 ± 0.82	−67.69 ± 1.10

## Data Availability

Data sharing not applicable.

## Sample Availability

Samples of the proteins presented in this study are available from the authors.
